# *Lactobacillus paracasei*-induced lung abscess in a splenectomized patient: A rare case report

**DOI:** 10.1097/MD.0000000000043162

**Published:** 2025-07-11

**Authors:** Zhimin Xiao, Yan Gu

**Affiliations:** a Department of Respiratory and Critical Care Medicine, The Affiliated Hospital of Inner Mongolia Medical University, Hohhot, Inner Mongolia, China.

**Keywords:** after splenectomy, immunocompromised host, *Lactobacillus paracasei*, lung abscess, multidisciplinary collaboration, next-generation sequencing, opportunistic infection, probiotic-related infection

## Abstract

**Rationale:**

*Lactobacillus paracasei* (LP), a probiotic species, is rarely associated with lung abscesses. This case highlights its role as an opportunistic pathogen in immunocompromised patients.

**Patient concerns:**

A 65-year-old splenectomized man presented with persistent abdominal pain, fever, and respiratory symptoms. Imaging revealed a right lung abscess, mediastinal lymphadenopathy, and adrenal nodules. Next-generation sequencing (NGS) confirmed LP infection. Multidisciplinary management with tailored antibiotics (faropenem) led to clinical improvement.

**Diagnoses:**

Comprehensive examination, including imaging studies, revealed a right lung abscess complicated by mediastinal lymphadenopathy, bilateral pleural effusion, and adrenal nodules. Subsequent lung puncture biopsy and NGS testing confirmed LP infection and the presence of an accessory spleen.

**Interventions:**

In collaboration with a multi-disciplinary treatment, the patient’s antibiotic treatment plan was adjusted based on the confirmed diagnosis of LP infection. Management included targeted antibiotic therapy (faropenem) tailored to the identified pathogen.

**Outcomes:**

The patient’s condition gradually improved following the adjusted treatment plan, leading to a reduction in symptoms and successful discharge from the hospital, with favorable follow-up results.

**Lessons:**

This case highlights LP as an emerging opportunistic pathogen causing severe lung abscesses in splenectomized patients. Early diagnosis via NGS and targeted antibiotic therapy guided by multidisciplinary collaboration were pivotal in achieving clinical resolution. Our findings underscore the importance of pathogen-specific management in immunocompromised hosts.

## 1. Introduction

*Lactobacillus paracasei* (LP) is a rod-shaped gram-positive bacterium from the *Lactobacillus* genus that is commonly used as a probiotic.^[1]^ Recently, the medical community has taken more interest in LP, although cases linking it to lung abscesses are rare. Lung abscesses occur when pus accumulates in the lungs because of microbial infections, often affecting individuals with weakened immune systems. Typical causes include aspiration, secondary infections, and bloodstream infections. The clinical manifestations of lung abscesses include fever, cough, and chest pain.^[2,3]^ Without prompt treatment, these conditions can lead to chronic complications. Under specific circumstances, LP can act as an opportunistic pathogen, causing severe infections, particularly in individuals with weakened immune systems, preexisting health conditions, or other risk factors.^[4]^

## 2. Case description

A 62-year-old male was admitted to the hospital for abdominal pain lasting 40 days, accompanied by fever, cough, and sputum for the past 5 days. Forty days before admission, he experienced persistent dull pain around his belly button for no apparent reason, which was initially ignored. Five days before admission, he suddenly developed a high fever of 39.5°C accompanied by chills and shivers, frequent coughing of thick yellow mucus, and sensations of chest tightness and shortness of breath. After 2 days of ineffective cephalosporin treatment at a local clinic, the patient decided to go to a local hospital. Chest CT revealed right lung inflammation, changes consistent with chronic bronchitis, and right pleural effusion. Abdominal CT indicated the presence of a right kidney cyst abdominal aortic atherosclerosis, and right pleural effusion. The white blood cell count (WBC) was 21.89 × 10^9^/L, the neutrophil count (NC) was 18.32 × 10^9^/L, the neutrophil percentage was 83.7%, the procalcitonin level was 6.97 ng/mL, the C-reactive protein (CRP) level was 127.44 mg/L, the alanine aminotransferase (ALT) level was 49 U/L, the aspartate aminotransferase (AST) level was 45 U/L, the albumin (ALB) level was 32.60 g/L, the blood glucose level was 7.00 mmol/L, and the sodium level was 129 mmol/L. Additionally, both the COVID-19 nucleic acid and antibody tests were negative. The local hospital provided several treatments including oral zinc cloth granules, intramuscular dexamethasone, intravenous methylprednisolone (40 mg), cefoperazone/sulbactam, imipenem for anti-inflammatory therapy, nebulization, and fluid replacement for symptom relief. The patient’s temperature initially returned to normal, but it rose again 2 hours later, peaking at 38.4°C. In addition, the patient experienced increased sputum production and worsening dyspnea. During the illness, the patient exhibited poor mental status, loss of appetite, and fatigue and no significant change in weight. On September 29, 2020, the patient was transferred to our hospital for treatment. Upon admission, pulmonary infection with pleural effusion was suspected. The nature of the right upper lung lesion and cause of abdominal pain are still under investigation. Physical examination revealed dullness to percussion in the lower field of the right lung, tremor, weakened breath sounds, and moist rales in both lungs. The abdomen was soft, with surgical scars on the left abdomen and tenderness around the umbilicus, left upper quadrant, and right lower quadrant, but no rebound tenderness.

**Figure 1.**
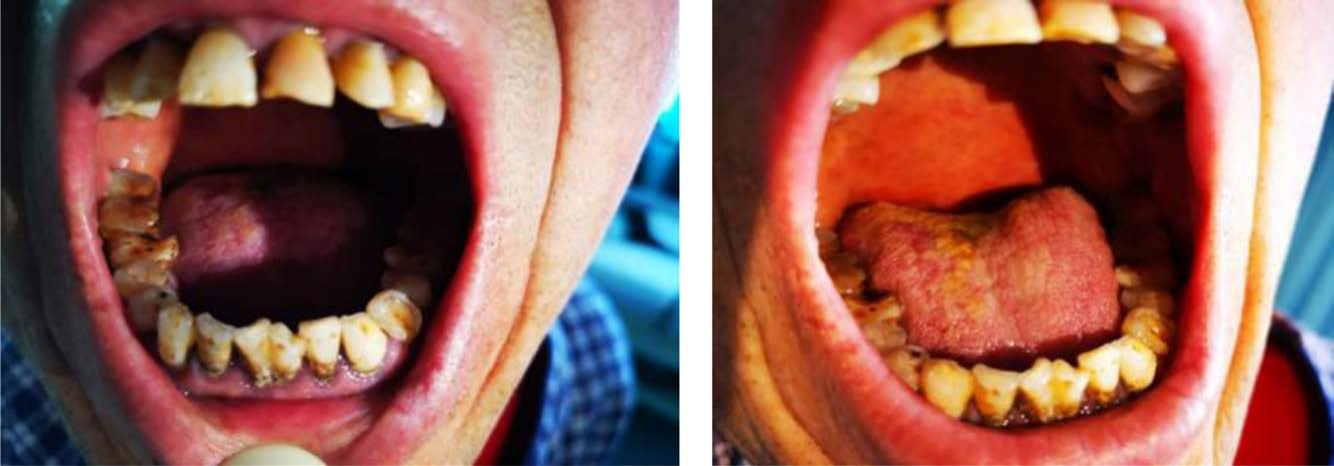
Oral hygiene status of the patient.

The patient had a 2-year history of hypertension with irregular treatment and poor compliance to monitoring. The highest recorded blood pressure was 160/130 mm Hg. The patient also underwent splenectomy due to traumatic splenic rupture. Personal medical history included long-term smoking and alcohol consumption, specifically 20 cigarettes/d for 40 years and 400 to 500 g of alcohol daily for 30 years. The patient had poor oral hygiene (Fig. 1) and denied any known family history of genetic disease. Furthermore, there was no reported history of contact with special infectious diseases or allergies, except for a known allergy to penicillin.

**Figure 2.**
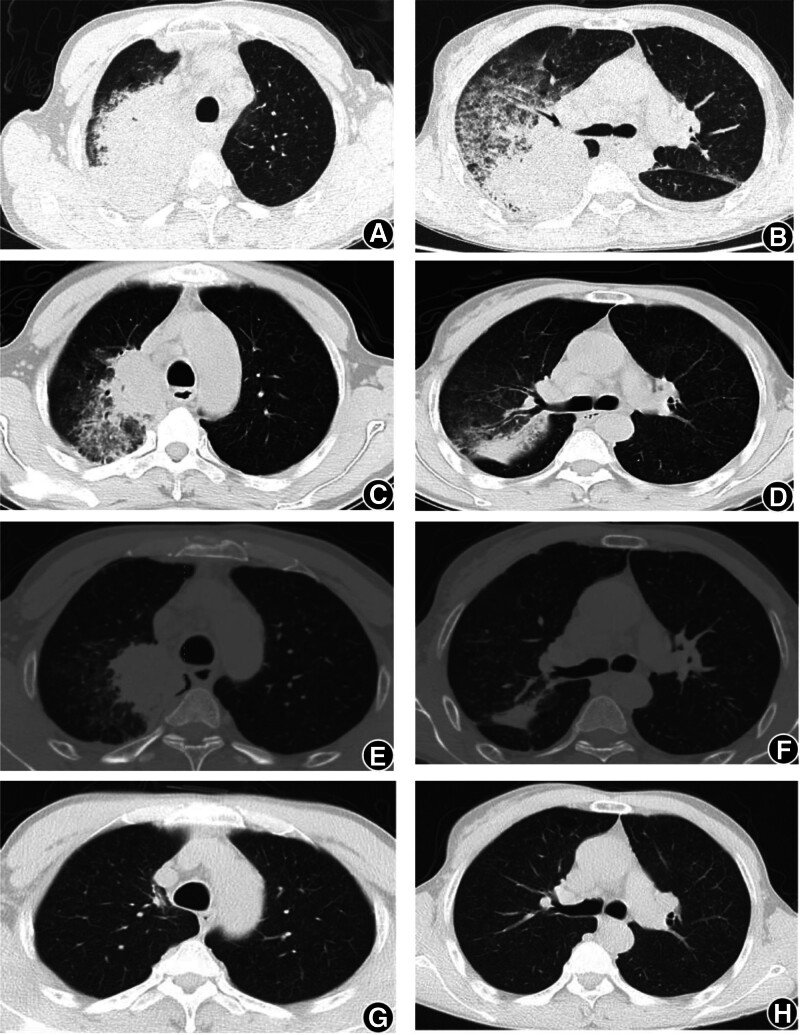
The chest CT scan findings for the patient: (A, B) dated September 30, 2020; (C, D) dated October 12, 2020; (E, F) dated October 22, 2020; and (G, H) dated July 19, 2021.

After admission, arterial blood gas analysis (in a non-oxygen state): pH 7.48, PaCO_2_ 28.7 mm Hg, PaO_2_ 57 mm Hg, SaO_2_ 91.6%, oxygenation index is <300. Blood tests revealed the following results: WBC 29.19 × 10^9^/L, NC 18.98 × 10^9^/L, CRP level, 194.26 mg/L, ALT 114 U/L, AST 155 U/L, ALB 29.3 g/L, and blood glucose 6.70 mmol/L. Infection-related tests revealed the following results: blood culture was negative, Legionella pneumophila culture was negative, sputum test for *Pneumocystis jirovecii* was negative, *Mycoplasma pneumoniae* IgG antibody test was positive, Brucella antibody was negative, and the infection panel was negative. However, sputum smears and cultures revealed the presence of *Candida krusei* and yeast like spores. Fungal culture of the lung tissue samples was negative. In contrast, chest CT (Fig. 2A, B) revealed right lung consolidation and enlarged mediastinal lymph nodes, along with inflammation in both lungs and bilateral pleural effusions. Abdominal computed tomography (CT) revealed a small nodule in the right adrenal gland, while the spleen was not visible. In addition, multiple nodules were observed in the splenic region, which may indicate the presence of an accessory spleen.

On admission, the preliminary diagnosis included double pneumonia, Type I respiratory failure, liver dysfunction, and hypoproteinemia. The nature of the right upper lung lesion is still being determined and the characteristics of the right adrenal nodule are under investigation. Additionally, abdominal pain requires further evaluation, and the patient is classified as having stage 3 hypertension (high-risk category) and impaired glucose tolerance.

**Figure 3.**
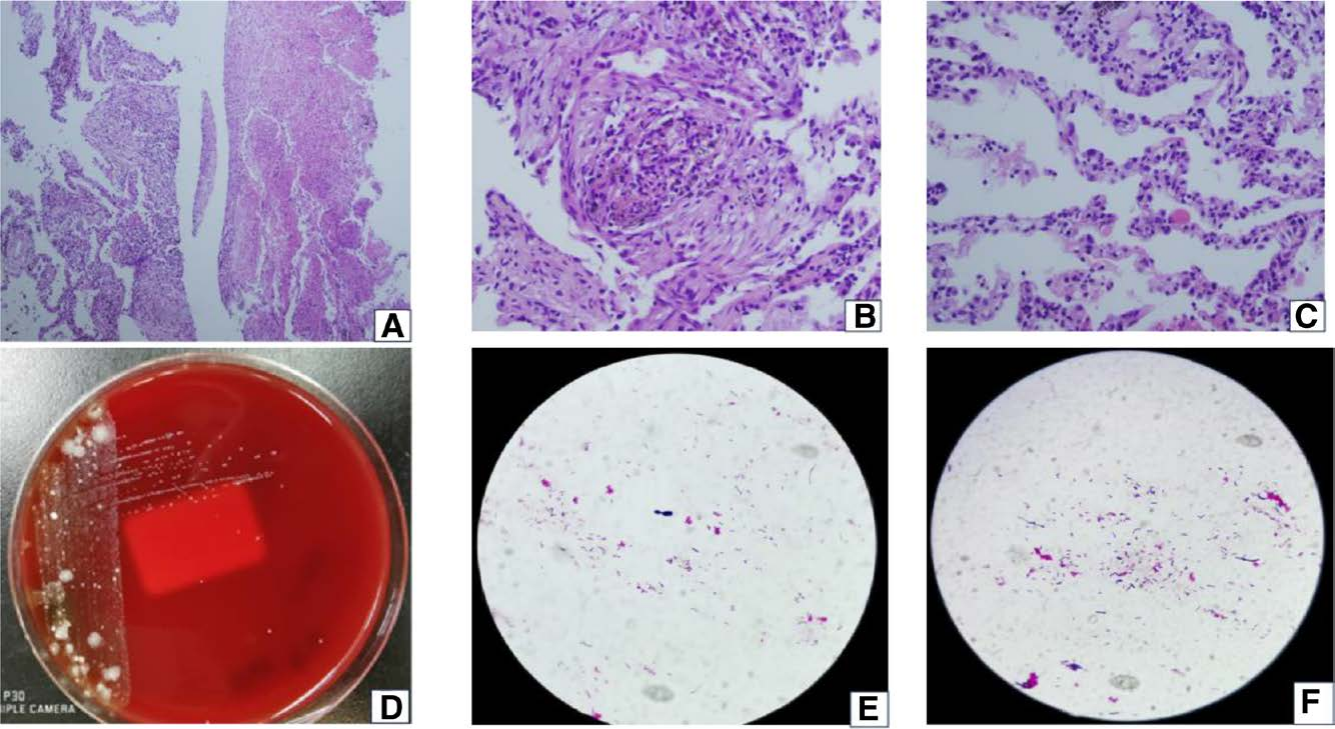
(A) Pathological examination of lung biopsy: fibrin exudation, chronic inflammatory cell infiltration and fibrous connective tissue hyperplasia (HE×100 times); (B) pathological examination of lung biopsy: fibrous connective tissue hyperplasia with small abscess formation (HE×200 times); (C) Lung biopsy pathology examination: chronic inflammatory cell infiltration in the alveolar septa, and partial alveolar septal rupture (HE×200 times); (D) blood agar plate with *Lactobacillus* colonies; and (E, F) *Lactobacillus* under the microscope. HE = hematoxylin and eosin.

**Figure 4.**
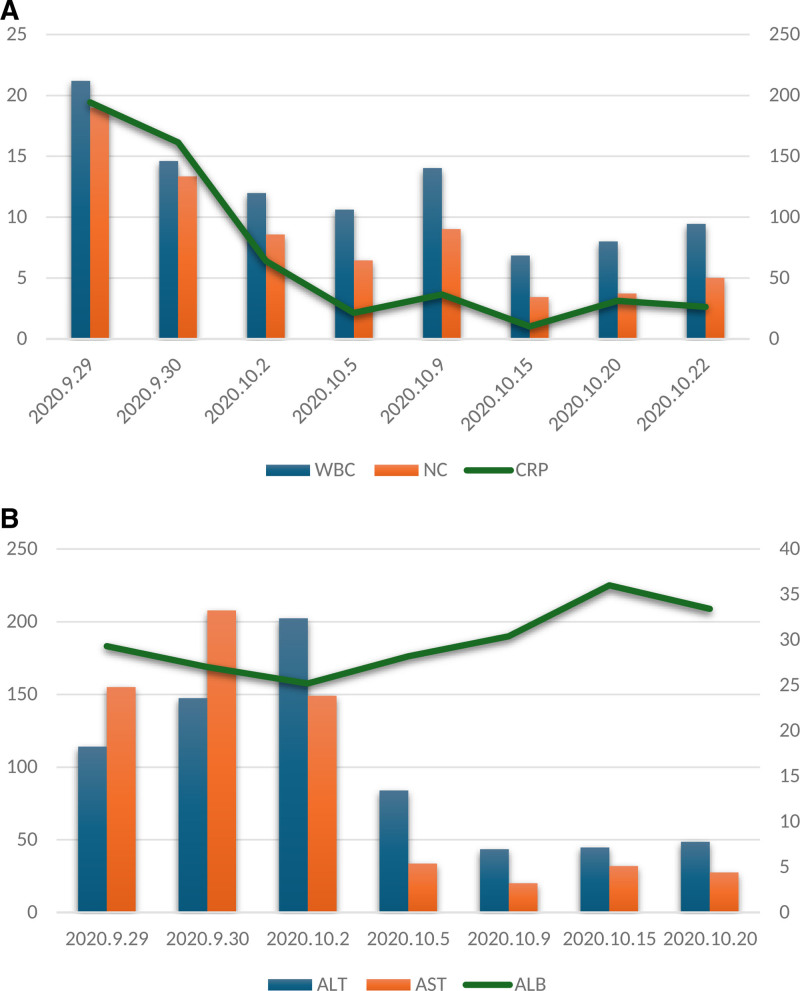
(A) Trend chart of changes in inflammatory indicators during treatment. (B) Trend chart of changes in liver function during treatment.

Treatment process:

1. Initial treatment (September 29–30): The patient presented with fever, maximum temperature of 38.4℃, difficulty breathing, headache, cough, phlegm, chest pain, and abdominal pain. Arterial blood gas analysis revealed a PaO_2_ of 47 mm Hg. The treatment included Meropenem 1.0 g every 8 hours and Moxifloxacin 0.4 g once a day to fight the infection, and glutathione 1.8 g once a day to help with abnormal liver function, along with symptomatic treatments such as antipyretics, fluid replacement, and nebulization.
2. Fluctuating condition (October 1–3): The patient’s temperature ranged from 37.2℃ to 38.2℃ (from the first to the third), with persistent difficulty breathing and high-flow oxygen therapy at 5 L/min using a mask; however, there was no improvement in oxygen levels (SaO_2_ 90%). The patient produced a lot of yellow-red sputum (approximately 50 mL/d). Chest ultrasound showed pleural effusion, with 150 mL on the right and 140 mL on the left side. Since there was no significant improvement, noninvasive ventilation was added, and infection treatment was continued.
3. Pathogen examination and treatment adjustment (October 4–11): On October 4, the patient experienced shortness of breath and a lot of phlegm, frequent nocturnal coughing, pain in the right chest and abdomen, maximum temperature of 36.8℃, ALB 25.2 g/L, and protein supplementation. On October 5, the antibiotics were adjusted; moxifloxacin was discontinued and replaced with teicoplanin 0.4 g once daily. Thoracentesis drainage was performed (ultrasound showed left 500 mL, right 1500 mL), with approximately 1350 mL of fluid drained from the right thoracic cavity. Routine examination of pleural fluid showed Li Fan positive ↑, polymorphonuclear cells 33.00%, mononuclear cells 67.00%, cell count 2912.00/mm^3^; biochemical analysis of pleural fluid: TPR 29.3 g/L, ADA 22.1 U/L, LDH 411 U/L, and no cancer cells were found in the pleural fluid sent for examination. They added 40 mg of methylprednisolone (October 4–6), and the patient’s temperature gradually stabilized, respiratory distress improved, hypoxia corrected, and abdominal pain alleviated. A routine blood and biochemical examination on October 5 showed: WBC 10.62 × 10^9^/L, NC 6.45 × 10^9^/L, CRP 21.34 mg/L, ALT 83.9 U/L, AST 33.6 U/L. On October 7, noninvasive ventilation was discontinued, and high-flow nasal oxygen therapy was initiated. By October 8, the patient’s temperature had been normal for 3 days, no fluid drainage was needed, the patient had minimal sputum production, no fever or abdominal pain, breath sounds in the right lung were enhanced, and the thoracic drainage tube was removed.
4. Further examination and treatment (October 12–17): On October 12, follow-up chest CT (Fig. 2C, D) showed a mass-like soft tissue density shadow in the posterior segment of the right upper lobe with unclear borders. Bilateral hilar enlargement and multiple enlarged mediastinal lymph nodes were observed. They suspected central lung cancer in the right lung with obstructive inflammation in the upper right lobe, mediastinal lymph node metastasis, bilateral pleural effusion, T9 nodular high-density shadow, metastasis to the right adrenal gland, and suspected metastasis. On October 13, the patient had no fever, intermittent cough, or resolved lung rales. Bronchoscopy revealed no abnormalities in either side of the bronchial mucosa or lumen. No tumor cells were detected on brush biopsy. On October 14, the patient had no fever, chest or abdominal pain, just a little cough, or yellow sputum, and the lung rales resolved. They discontinued meropenem and teicoplanin, replacing them with etimicin (200 mg once daily and cefepime 2.0 g every 12 hours) . That evening, a CT-guided biopsy of the right lung was performed, and tissue samples were sent for culture (bacteria, fungi, and TB smear) and acid-fast staining, all of which were negative. Biopsy pathology indicated chronic suppurative inflammation with abscess formation, focal organization pneumonia changes, and alveolar epithelial hyperplasia (Fig. 3A–C). On October 15, laboratory tests showed: WBC 6.85 × 10^9^/L, NC 3.44 × 10^9^/L, CRP 10.3 mg/L, ALT 44.7 U/L, AST 31.9 U/L. During this period, the patient’s condition stabilized, cough significantly improved, with almost no sputum production and no shortness of breath or chest pain discomfort.
5. Recurrence of the condition (October 18–20): On October 18, the patient again experienced fever (*T*_max_ 38.0℃), and symptoms of abdominal pain and pain in the right chest and back reappeared. Communication with the family led to the submission of Next-generation sequencing (NGS), which indicated the presence of *Lactobacillus* (sequence number 204206) and LP (sequence number 166825). From October 19 to 20, *T*_max_ was 37.9℃, with dizziness and headache following the temperature rise, and dull pain around the navel and right abdomen. Physical examination revealed a soft neck, right lung base with scattered moist rales on auscultation, tenderness (+) around the navel and right lower abdomen, and bilateral upper abdominal tenderness. Etimicin was discontinued, and linezolid was started at 600 mg every 12 hours.
6. Multidisciplinary consultation and subsequent treatment (October 21–29): On October 21, the patient occasionally had a dry cough, no sputum, no abdominal pain or chest pain discomfort, and *T*_max_ 37.1℃. The bacteriology laboratory rechecked the sputum and tissue culture results (Fig. 3D–F), confirming the growth of a large number of gram-positive bacilli, identified by mass spectrometry as LP, which was resistant to vancomycin. PET-CT examination showed inflammatory proliferative lesions in the right upper lobe with central liquefactive necrosis (suspected lung abscess or granulomatous inflammation), distal lung atelectasis, and obstructive inflammation. Compared to the lung CT on September 29, 2020, the mass volume had decreased (Fig. 2E, F), multiple lymph nodes showed inflammatory hyperplasia, and there was no significant change in the size of the lymph nodes behind the trachea; the right pleural effusion had significantly reduced; both lungs had old lesions, pulmonary bullae, and a small amount of right pleural effusion; 2 rounded shadows were observed in the left abdominal cavity, likely residual tissue after splenectomy; a mid-pole cyst in the right kidney; and a strip of soft tissue shadow in the ileocecal region with increased metabolism and suspected appendicitis. Multidisciplinary expert consultation: Based on the patient’s clinical symptoms, imaging studies, and pathogen examination, the right lung abscess appeared as consolidation in the right upper lobe, with pus accumulation and mediastinal lymph node enlargement. Chest CT and thoracentesis examination results (the tissue samples showed a high bacterial load in the affected area, and sputum culture showed a large number of G^**+**^ bacilli) and NGS indicated LP, further supporting the diagnosis of lung abscess, and tumor lesions were not considered at this time. They thought that the left upper abdominal lesion was probably leftover tissue from the spleen, and gastrointestinal endoscopy and upper abdominal MRI were performed to exclude other abdominal lesions. Gastroscopy indicated a fundal bulge (possible stromal tumor) and chronic atrophic gastritis. Upper abdominal MRI suggested a splenic remnant and right adrenal nodule. Follow-up blood routine and biochemical tests showed significant decreases compared to before (Fig. 4A, B): white blood cells 9.45 × 10^9^/L, NC 5.02 × 10^9^/L, CRP 26.49 mg/L, ALT 48.6 U/L, AST 27.5 U/L, ALB 33.40 g/L. Intravenous medications were discontinued, and oral faropenem (200 mg 3 times a day) was administered. The patient felt fine, had a dry cough, had no sputum, and their condition stabilized. On October 29, the patient was discharged, continuing oral faropenem for 14 days for infection treatment, and underwent accessory splenectomy in the gastrointestinal surgery department 1 month later. Follow-up visits showed that they recovered well (Fig. 2G, H).

## 3. Discussion

This report presents a rare case of a complex lung abscess caused by LP. The uniqueness of this case is that the patient not only had multiple underlying diseases, but also underwent splenectomy. In cases of complex infections, lung abscesses caused by LP, an opportunistic pathogen, are relatively rare in clinical practice. Early identification of pathogens and implementation of precise treatment are essential, especially in patients with impaired immune function.

*Lactobacillus* is a major component of the microbial community in many fermented foods and is widely used as a probiotic. LP, a potential probiotic strain, has various health benefits. This strain can reduce oxidative damage, improve cognitive function, alleviate symptoms of colitis and allergic bronchitis, assist in the treatment of metabolic diseases (such as type 2 diabetes and hyperlipidemia), and inhibit cancer cell proliferation and promote apoptosis.^[5–11]^ These findings support the use of LP in health promotion and disease prevention. However, under specific conditions such as immunocompromised states, diabetes, absence of other underlying conditions, or presence of other risk factors (such as artificial heart valves, dental surgery, or dental caries), this bacterium can transform into an opportunistic pathogen, leading to severe infections.^[4]^ In recent years, several cases of clinical infections caused by LP have been reported (Table 1).

**Table 1:** Clinical cases of infection due to LP.

Infection type	Age	Gender	Underlying diseases	Treatment drugs	Duration	Treatment outcome
Liver abscess and *Lactobacillus paracasei* bacteremia^[12]^	65	Female	Type 2 diabetes, hypertension, and hyperlipidemia	(ICU) Piperacillin-tazobactam, metronidazole, and gentamicin, along with insulin therapy (after confirming LP in liver abscess culture) antibiotics switched to amoxicillin-clavulanate (persistent periodic high fever) antibiotics switched to linezolid, metronidazole, amikacin, and fluconazole	12 wk	Recovering well after receiving antibiotic change and successful drainage of abscess
Infective endocarditis^[13]^	47	Male	Heart disease and inguinal hernia	Piperacillin-tazobactam IV 2.25 g/12 h	3 wk	Clinical cure
Infective pancreatic necrosis and retroperitoneal abscess^[14]^	88	Female	Type 2 diabetes, hypertension, and dementia	(initial) Ampicillin and cefmetazole (new retroperitoneal abscess appeared) penicillin G (stable) oral amoxicillin	74 d	Clinical cure
Prosthetic joint infection after total hip arthroplasty^[15]^	82	Female	Left renal cell carcinoma treated with nephrectomy, asthma, hypertension, dyslipidemia, and hypothyroidism	(after initial Revision surgery) Ampicillin for 6 wk (after third initial revision surgery) ampicillin for 6 wk, with gentamicin for 3 d (after permanent resection arthroplasty) ampicillin for 6 wk, then transitioned to long-term oral amoxicillin	18 wk	Good recovery, no significant pain, improved function of the right hip joint
Prosthetic valve endocarditis^[16]^	65	Male	Rheumatic fever, mitral valve prolapse, and history of artificial heart valve surgery	(initially) Vancomycin and ceftriaxone (after obtaining drug sensitivity results) penicillin and gentamicin for 6 wk; then switched to oral penicillin, 500 mg 4 times/d	7 mo	Clinical condition stable, no plans for repeat aortic valve replacement

LP = *Lactobacillus paracasei*.

A lung abscess is a purulent inflammation of the lungs caused by microbial infection, often accompanied by symptoms such as fever, cough, and chest pain. This patient became a high-risk group for LP infection due to long-term smoking and drinking, poor oral hygiene, impaired immune function after splenectomy, and multiple underlying diseases, which made the lungs susceptible to invasion and development of lung abscesses. Its clinical manifestations are complex, with abdominal pain as the main symptom in the early stage, followed by respiratory symptoms, such as high fever, cough, sputum production, and chest pain. It is easily misdiagnosed as pneumonia or lung cancer at initial diagnosis. Although persistent abdominal pain symptoms are not directly related to respiratory symptoms, they are considered to be retroperitoneal inflammation or distant metastasis caused by a lung abscess, which increases the difficulty of diagnosis. The patient underwent multiple imaging examinations, lung puncture, and NGS combined with bacterial culture to confirm the diagnosis of a lung abscess caused by LP infection. In addition, the patient’s lung abscess combined with pleural effusion further increased the complexity of treatment. There is a lack of clear optimal treatment plans for the treatment of lung abscesses caused by LP due to the variable sensitivity of bacterial species and the complexity of identification. According to the literature,^[1]^ the most commonly used antibiotics include penicillins (penicillin and ampicillin) or combined aminoglycosides; however, the vancomycin resistance rate is high (only 22.5% of cases are susceptible). However, the anti-infective treatment for this patient has undergone many adjustments, from the initial use of carbapenems combined with fluoroquinolones (meropenem and moxifloxacin) to the final use of faropenem after LP infection was determined. The treatment was also changed. Remarkable clinical results were obtained in this study. Clinical challenges in managing LP infections are well-documented, particularly regarding pathogen identification and the complexity of anti-infective regimens. In this case, the patient was not effectively controlled in early treatment, suggesting that clinicians should remain highly vigilant when facing such rare infections and conduct timely etiological examinations and imaging evaluations to adjust treatment plans as early as possible. By applying NGS technology, we can identify LP infections more quickly and adjust anti-infection strategies accordingly, thereby improving patient prognosis.

The successful treatment of this patient depended on an multi-disciplinary treatment that included experts from various fields, such as Respiratory and Critical Care Medicine, Infectious Diseases, Gastroenterology, Thoracic Surgery, Laboratory Medicine, Radiology, and Pathology. This improved the diagnostic accuracy and ensured that the treatment plan was both specific and effective. After active treatment, the patient’s condition stabilized, and they were discharged; however, we need long-term follow-up to monitor changes in the patient’s condition and prevent recurrence, particularly focusing on how the pulmonary lesions are absorbed and any changes in the accessory spleen. Considering the various underlying diseases and risk factors, including hypertension, long history of smoking and drinking, and poor oral hygiene, appropriate health education and interventions should be implemented to reduce the risk of reinfection.

## 4. Conclusion

This case illustrates the potential virulence of LP in immunocompromised individuals and advocates for NGS-guided diagnostics in complex infections. Clinicians should maintain a high index of suspicion for rare pathogens in splenectomized patients with atypical presentations. Further studies are needed to establish standardized treatment protocols for LP-associated infections.

## Acknowledgments

The authors thank the patient and his relatives for agreeing to report the case and providing a detailed medical history.

## Author contributions

**Conceptualization:** Zhimin Xiao.

**Data curation:** Zhimin Xiao.

**Formal analysis:** Zhimin Xiao.

**Investigation:** Zhimin Xiao.

**Supervision:** Yan Gu.

**Writing – original draft:** Zhimin Xiao.

**Writing – review & editing:** Zhimin Xiao, Yan Gu.
